# Hyponatremia in elderly patients with fragility fractures of the proximal femur: a cross-sectional study

**DOI:** 10.1590/2175-8239-JBN-2019-0019

**Published:** 2019-08-19

**Authors:** Aída Fernanda Batista Rocha, Marcus Villander Barros De Oliveira Sá, Ubirace Fernando Elihimas

**Affiliations:** 1 Universidade Federal de Pernambuco, Hospital das Clínicas, Recife, PE, Brasil.; 2 Real Hospital Português, Recife, PE, Brasil.; 3 Real Hospital Português, Unidade de Nefrologia, Recife, PE, Brasil.

**Keywords:** Hyponatremia, Fractures, Bone, Femur, Aged, Hiponatremia, Fraturas Ósseas, Fêmur, Idoso

## Abstract

**Introduction:**

Proximal femur fractures affect the mortality and morbidity of elderly individuals. Recent studies have shown an association between fragility fractures and hyponatremia, a common fluid and electrolyte balance disorder.

**Objectives:**

This study aimed to investigate the occurrence of hyponatremia in patients with fragility fractures of the proximal femur.

**Methods:**

The authors looked into the data from the medical records of patients admitted to the emergency unit of the Real Hospital Português for fragility fractures of the proximal femur from 2014 to 2017. The study included patients with serum sodium levels recorded in their charts.

**Results:**

Fourteen of 69 (20.3%) patients with proximal femur fractures had hyponatremia. The main factors linked to hyponatremia were lung disease, and prescription of amiodarone and/or antidepressants.

**Conclusion:**

In elderly individuals, fragility fractures of the proximal femur may correlate with hyponatremia, particularly among patients on amiodarone or antidepressants.

## RESUMO

**Introdução:**

Fratura de fêmur proximal tem impacto na mortalidade e morbidade de idosos. Estudos recentes vêm demonstrando associação entre fratura por fragilidade e hiponatremia, um distúrbio hidroeletrolítico comum na prática médica.

**Objetivos:**

Investigar a ocorrência de hiponatremia em pacientes com fratura proximal de fêmur por fragilidade.

**Metodologia:**

Foram coletados dados a partir de prontuários de pacientes admitidos na emergência do Real Hospital Português devido à fratura proximal de fêmur por fragilidade, entre 2014 e 2017, e aqueles com natremia disponível no prontuário eletrônico foram incluídos no estudo.

**Resultado:**

Dentre os 69 pacientes com fratura de fêmur proximal, houve uma ocorrência de 14 pacientes com hiponatremia, o que corresponde a 20,3%. Os principais fatores associados à hiponatremia no estudo foram doença pulmonar, uso de amiodarona e antidepressivos.

**Conclusão:**

Em idosos, a fratura de fêmur proximal por fragilidade pode estar correlacionada com hiponatremia, principalmente quando estão sob uso de amiodarona ou antidepressivos.

## INTRODUCTION

Hyponatremia is the most common fluid and electrolyte balance disorder.^1^ Some authors have described a correlation between hyponatremia and proximal femur fractures.^2^^,^ 3 ^,^ 4 Patients with asymptomatic hyponatremia are at higher risk of falling on account of gait disorders.^5^ Additionally, hyponatremia may also stem from decreased bone mineral density secondary to osteoclast activation.^2^ In 2011, Barsoni et al. submitted that hyponatremia might induce oxidative stress on osteoclasts, in a proposition known as the theory of oxidative stress on ascorbic acid transporters.^6^ In 2016, Fibbi et al. described hyponatremia as a regulator of the expression of genes MCP-1 (*Monocyte chemoattractant protein-1*) and CXCL-12 (*C-X-C Motif Chemokine Ligand 12*) connected to osteoclastogenesis - the osteoclastogenesis modulation theory.^7^

The etiology of hyponatremia must be defined before specific medical treatment is prescribed.^8^ Hyponatremia may be categorized based on the volume status of the patient as hypovolemic, euvolemic, or hypervolemic.^9^ Syndrome of inappropriate secretion of antidiuretic hormone (SIADH) ranks as one of the main causes of euvolemic hyponatremia.^8^ SIADH may occur secondarily to malignant lung, mediastinal, gastrointestinal, and genitourinary tumors. SIADH may originate from asthma, cystic fibrosis, chronic obstructive pulmonary disease (COPD), and infectious lung diseases such as viral or bacterial pneumonias, tuberculosis, aspergillosis, and lung abscesses. SIADH may be caused by other neurologic factors including vascular malformations, mass lesions, stroke, head trauma, and infections such as encephalitis, meningitis, malaria, acquired immunodeficiency syndrome, and brain abscesses. SIADH may still occur in association with the use of medications such as vasopressin analogues, antidepressants, antipsychotics, anticonvulsants, cancer drugs, opioids, proton pump inhibitors, amiodarone, and non-steroid anti-inflammatory drugs.^10^

Falling is a common incident among the elderly that impacts their morbidity and mortality. An estimated 30% of the individuals aged 65+ years fall every year (Yale Health Project 1988),^11^ an event responsible for approximately 5% of the hospitalizations of elderly persons.^12^ Hip fractures are placed among the most severe consequences of falling, with reported one-year mortality ranging between 26% and 33%.^13^ Several instruments have been developed to identify individuals at risk of fracture, such as the Fracture Risk Assessment Tool (FRAX) of the World Heath Organization published in 2008,^14^ and the QFracture algorithm developed for the British population. Since fractures significantly affect the quality of life of elderly individuals and impose a sizable financial burden on healthcare systems, it is only fitting to recognize, prevent, and address risk factors such as hyponatremia. This study aimed to investigate the occurrence of hyponatremia in patients with fragility fractures of the proximal femur.

## METHODS

This cross-sectional study was carried out at a tertiary referral hospital in the Brazilian city of Recife to investigate the occurrence of hyponatremia in patients with proximal femur fractures.

The study enrolled elderly patients (individuals with ages ≥ 60 years, as per the definition of the Brazilian Ministry of Health)^15^ hospitalized for proximal femur fractures (femoral neck, transtrochanteric, or subtrochanteric fractures)^16^ caused by falls from standing height or less that would not result in fractured bones in the majority of healthy individuals (fragility or low energy fractures).^14^ Additionally, included patients were required to have serum sodium levels recorded in their charts at the time of admission to the emergency unit or at some point in the three months preceding hospitalization.

The patients were analyzed based on the following data: age; sex; serum sodium levels (mild hyponatremia < 135 mmol/L; moderate hyponatremia < 130 mmol/L; or severe hyponatremia < 125 mmol/L);^17^ prescribed medication (amiodarone, benzodiazepines, antidepressants, anticonvulsants, antipsychotics, diuretics, proton pump inhibitors); and comorbidities (systemic hypertension, diabetes mellitus, lung disease, hypothyroidism, heart failure, osteoporosis, history of stroke, dementia, history of bone fracture).

The authors examined the data collected from the charts of patients seen at the emergency unit of the Real Hospital Português from January 1, 2014 to December 31, 2017. The subjects included in the study were divided into two groups based on their serum sodium levels. Group 1 featured patients with hyponatremia (serum sodium < 135 mmol/L), while Group 2 enrolled individuals without hyponatremia (serum sodium ≥ 135 mmol/L). Statistical analyses were performed on software package R-project 3.4.2. Bartlett’s test was used to test the homogeneity of variances. Distribution normality was assessed via the Shapiro-Wilk test. Categorical variables were expressed as frequencies and proportions. Fisher’s exact test was used to evaluate the association between categorical variables and hyponatremia, and p-values < 0.05 were deemed significant. Numerical variables were expressed as mean values ± standard error (SE). Statistical differences between the groups with and without hyponatremia were analyzed with the aid of the Mann-Whitney U test and significance was attributed to differences with a *p*-value < 0.05.

The Research Ethics Committee of the University of Pernambuco approved this study and assigned it permit CAE 94620518.7.0000.5207 on November 6, 2018.

## RESULTS

**Figure 1:**
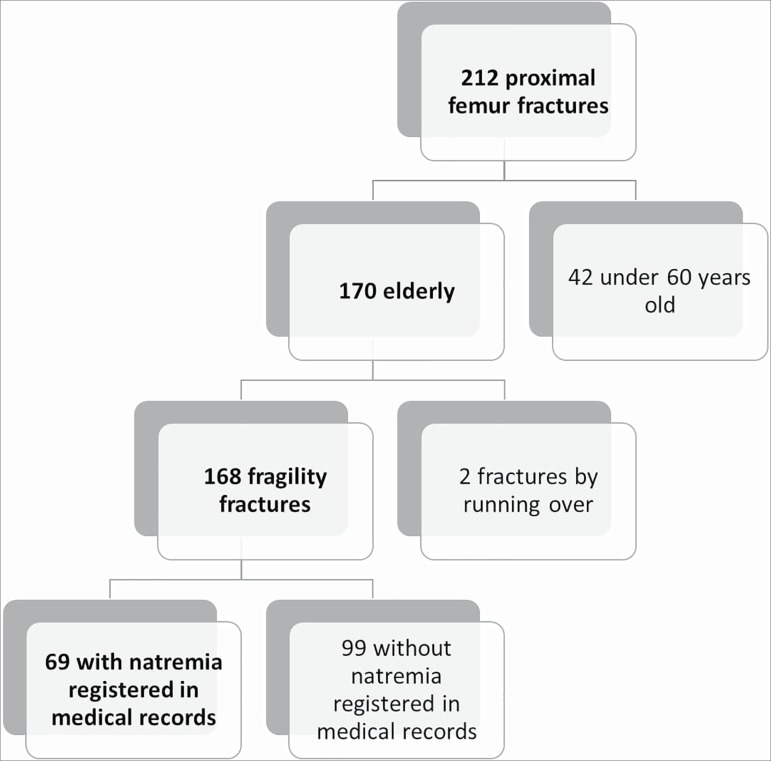
Patient selection flowchart

A total of 212 patients were admitted to the emergency unit of the Real Hospital Português from January 1, 2014 to December 31, 2017 for proximal femur fractures. Forty-two were excluded for having ages of less than 60 years. Two patients with bone fractures for being hit by automobiles were also excluded. Ninety-nine were excluded for not having their serum sodium levels measured at admission or in the three months preceding hospitalization for a fractured femur. Therefore, the study population added up to 69 patients (Figure 1).

Nearly four fifths (79.7%) of the 69 included patients did not have hyponatremia. In the group with hyponatremia (20.3%), mild, moderate, and severe hyponatremia was seen in 78.6%, 14.3%, and 7.1% of the patients, respectively.

Table 1 shows that there was no statistically significant difference in the sex (*p* = 0.527) of age (*p* = 0.317) distributions of the two groups.

**Table 1:** Characteristics of the patients with fragility fractures of the proximal femur in Groups 1 (serum Na < 135 mmol/L) and 2 (serum Na ≥ 135 mmol/L) admitted to the emergency unit of the Real Hospital Português from January 1, 2014 to December 31, 2017.

Variables	Group 1	Group 2	*p-value*
	< 135 mmol/L	≥ 135 mmol/L	
	(N = 14)	(N = 55)	
Serum sodium	131.1 ± 3.7 SE (120 - 134)	141.3 ± 4.3 SE (136 - 161)	< 0.001
**Sex**			0.527
Female	57.1%	69.1%	
Male	42.9%	30.9%	
Age	83.9 ± 7 DP (67 - 95)	82 ± 7.8 DP (63 - 100)	0.317

* Note: the serum sodium levels considered were the most recent in the three months leading to hospitalization or the levels measured at admission

The mean serum sodium level seen in Group 1 was statistically different from the level

Table 2 lists the comorbidities observed in the two groups of patients and shows that the individuals in Group 1 had lower serum sodium levels and significantly greater involvement by lung disease (*p* = 0.034). Three quarters of the patients with lung disease in Group 1 had COPD and one quarter was diagnosed with idiopathic pulmonary fibrosis. A third of the patients with lung disease in Group 2 had asthma, a third had COPD, and a third was diagnosed with bronchiectasis.

**Table 2:** Comorbidity analysis - Group 1 vs. Group 2. Elderly patients with fragility fractures of the proximal femur admitted to the emergency unit of the Real Hospital Português from January 1, 2014 to December 31, 2017.

Comorbidities			*p-value*
	Na < 135 mmol/L	Na ≥ 135mmol/L	
	(N = 14)	(N = 51)*	
Hypothyroidism	21.40%	3.90%	0.063
Systemic hypertension	71.40%	68.60%	1
Coronary artery disease	0.00%	2.00%	1
Osteoporosis	21.40%	21.60%	1
Diabetes mellitus	21.40%	33.30%	0.521
Dementia	28.60%	21.60%	0.721
Lung disease	28.60%	5.90%	0.034
History of fragility fracture	0.00%	5.90%	1
Cancer	0.00%	9.80%	0.576
Without bone metastases	0.00%	60.00%	
With bone metastases	0.00%	40.00%	
Heart failure	21.40%	15. 7%	0.691
History of stroke	0.00%	11.80%	0.327

* Note: four patients were excluded for having incomplete data.

**Figure 2:**
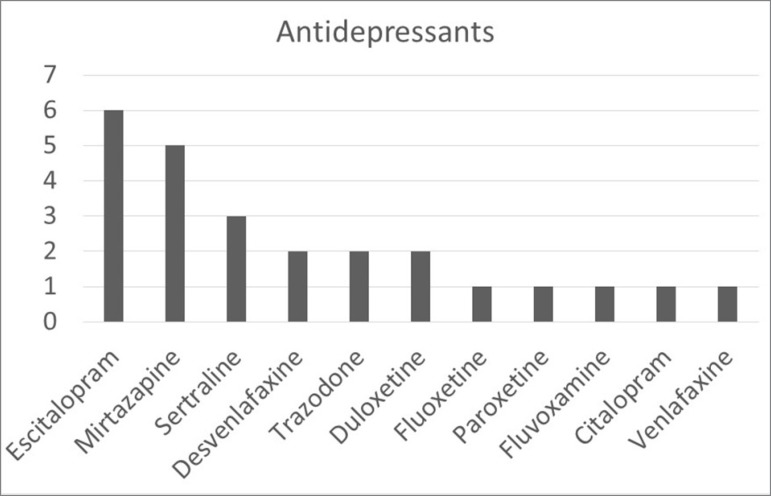
Number of patients in each of the prescribed antidepressants.

Statistical analysis indicated the existence of a correlation between hyponatremia and amiodarone (*p* = 0.007) and hyponatremia and prescription of antidepressants (*p* = 0.042), as seen in Table 3. The patients included in the study were on the following antidepressants: escitalopram (25.00%), mirtazapine (20.83%), sertraline (12.50%), desvenlafaxine (8.33%), trazodone (8.33%), duloxetine (8.33%), fluoxetine (4.17%), paroxetine (4.17%), fluvoxamine (4.17%), citalopram (4.17%), and venlafaxine (4.17%), as seen in Figure 2.

**Table 3:** A comparison between the medications prescribed to individuals in Groups 1 (serum Na < 135) and 2 (serum Na ≥ 135) admitted to the emergency unit of the Real Hospital Português for fragility fractures of the proximal femur from January 1, 2014 to December 31, 2017

Medication	Group 1	Group 2	*p*-*value*
	Na < 135 mmol/L	Na ≥ 135 mmol/L	
	(N = 14)	(N = 50)**	
Amiodarone	28.6%	2.0%	0.007
Diuretics	21.4%	26.0%	1.000
Benzodiazepines	28.6%	28.0%	1.000
Antidepressants	14.3%	44.0%	0.042
Anticonvulsants	7.1%	14.0%	0.673
Proton pump inhibitors	21.4%	22.0%	1.000
Antipsychotics	7.1%	16.0%	0.670

** Note: five patients were excluded for having incomplete data.

## DISCUSSION

This study aimed to investigate the occurrence of hyponatremia in patients with proximal femur fractures. Similarly to prior studies,^2^^-^ 4 ^,^ 18 ^-^ 22 fourteen (20.3%) of the 69 patients with proximal femur fractures had hyponatremia. The main findings associated with hyponatremia were lung disease and prescription of amiodarone and/or antidepressants.

Hyponatremia may be categorized based on the duration of the disorder as acute (< 48 hours) or chronic (> 48 hours).^23^ In chronic hyponatremia, the brain adjusts to the hypotonic environment and clinical manifestations are not quite as exuberant.^17^ Patients with chronic hyponatremia are generally asymptomatic or present with mild clinical anomalies such as attention deficit, gait disorders, falls, and impaired recovery from bone fractures.^24^^,^ 25

Upala and Sanguankeo published a meta-analysis featuring 12 trials and observed the existence of a significant association between bone fractures and osteoporosis in individuals with hyponatremia, with an odds ratio (OR) for fracture of 1.99 [95% confidence interval (CI); 1.50 - 2.63; *p* < 0.001] in studies reporting odds ratios, and increased relative risk (RR) of fracture of 1.62 (95%CI; 1.28 - 2.05; *p* < 0.001) in studies reporting risk measurements.^2^

Ayus et al. analyzed a retrospective cohort of 31,527 patients - 0.9% diagnosed with chronic hyponatremia - and found that the absolute risk of having a hip fracture was 3.07% in patients with chronic hyponatremia and 1.31% in individuals with normal serum sodium levels. Patients with hyponatremia had increased adjusted RR of having a hip fracture [4.52 (95%CI 2.14-9.6)]. The adjusted RR seen for the subset of patients with moderate hyponatremia (< 130 mmol/L) was even higher [7.61 (95%CI 2.8-20.5)].^3^

Gankam Kengne et al. looked into the prevalence of hyponatremia (serum sodium < 135 mmol/L) of 513 elderly patients with bone fractures and compared them against controls without fractures matched for sex and age. The prevalence of hyponatremia was significantly higher among individuals with bone fractures than controls (13% vs. 3.9%), with an adjusted OR of 4.16 (95%CI 2.24 - 7.71). In the cited study, hyponatremia was primarily caused by medication - diuretics (36%) and selective serotonin re-uptake inhibitors (17%) - and SIADH (37%).^18^

In a similar study, Sandhu et al. compared the incidence of hyponatremia in 364 elderly patients admitted to an emergency unit for bone fractures (hip/pelvis/femur) against another group of 364 elderly patients without bone fractures admitted to the same service within the same time period. The incidence of hyponatremia was significantly higher in the group with bone fractures (9.1% vs. 4.1%). The mean serum sodium level of the individuals in the fracture group was 131 ± 2 mEq/L. More than three quarters (75.3%) of the individuals in the bone fracture group were females. Nearly a quarter (24.2%) of the patients with bone fractures and hyponatremia were on antidepressants, 75% of which were selective serotonin re-uptake inhibitors (SSRIs). None of the patients in the fracture-free group was on antidepressants.^19^

Jamal et al. reviewed the data from the Osteoporotic Fractures in Men (MrOS) trial to elicit possible relationships between hyponatremia and bone fractures in 5,122 males aged 65 and older. The relative risk for hip fracture was 3.48 (95%CI: 1.76-6.87).^20^ Kinsella et al. looked into the data from 1,408 females submitted to bone densitometry and found that 18% had bone fractures. The incidence of hyponatremia was greater in the group with fractures (8.7%) than in the fracture-free group (3.2%).^21^

Rittenhouse et al. investigated 2,370 cases of trauma involving elderly individuals and found a prevalence of hyponatremia of 12.4% (OR: 1.81; 95%CI: 1.26-2.60; *p* = 0.001).^22^ Aicale et al. reported a prevalence of hyponatremia of 19% in a population of 334 elderly individuals with hip fractures.^4^

Amiodarone-induced hyponatremia is a rare complication, with only 17 cases described in the literature.^26^ The first case report was published in 1996.^27^ The mechanism by which amiodarone induces SIADH is still unclear.^28^ Iovino et al. suggested that amiodarone causes SIADH by stimulating anti-diuretic hormone secretion by the magnocellular neurons in the supraoptic and paraventricular nuclei of the hypothalamus or through the expression of water channel aquaporin-2 (AQP2) in the collecting ducts.^29^

More than a quarter (28.6%) of the individuals with hyponatremia included in our study were on amiodarone, suggesting that SIADH secondary to amiodarone prescription might not be that rare.

Prescription of antidepressants has been associated with increased risk of falling and fracture.^30^ Tricyclic antidepressants and mirtazapine have been linked to increased risk of falling on account of their effects in the ability to concentrate and balance, orthostatic hypotension, and sedative effects.^31^ SSRIs reportedly cause dizziness and increase the risk of fractures by decreasing bone mineral density.^32^

Macri et al. showed that subjects on antidepressants were at increased risk of falling when compared to individuals off antidepressants [5.2% vs. 2.8%; adjusted OR: 1.9, (95%CI: 1.7-2.2). The authors did not find statistically significant differences between antidepressant classes SSRIs, serotonin and norepinephrine reuptake inhibitors (SNRIs) and trazodone.^33^

Antidepressants increase the risk of falling via several different mechanisms,^30^ and have been considered to cause SIADH.^17^ The results of our study seem to indicate the existence of an association between antidepressants and proximal femur fractures (*p* = 0.042). However, we found more patients on antidepressants in the group with bone fractures without hyponatremia (44% of the subjects in Group 2 were on antidepressants vs. 14.3% in Group 1). A third (33.33%) of the 69 patients included in our study were on antidepressants. More than half (54.71%) of the patients included in our study were on SSRIs; 20.83% were on SNRIs; 20.83% were on mirtazapine; and 8.33% were on trazodone.

There seems to exist a relationship between hyponatremia and fragility fractures of the proximal femur in elderly patients, although recent studies^2^^-^ 4 have published new information connecting hyponatremia to falls^5^ and bone fractures.^6^ Serum sodium levels have been underreported for elderly patients admitted for falls. In the study period, only 69 of the 168 elderly individuals admitted for proximal femur fractures had serum sodium levels recorded in their charts - and a meager 45 had serum sodium levels measured upon admission.

Our study showed that amiodarone might be linked to hyponatremia. However, this inference can only be verified through a prospective study designed to establish a causal link between amiodarone and hyponatremia.

Lung diseases such as COPD and asthma or infectious conditions such as tuberculosis, aspergillosis, and bacterial pneumonia have been described as possible causes of SIADH.^34^ Some authors have described hyponatremia in COPD exacerbation.^35^^,^ 36 Although the mechanism by which hyponatremia occurs in COPD has not been fully elucidated, it has been suggested that hypercapnia decreases renal blood flow, thereby increasing the retention of water and sodium and causing edema and hyponatremia.^37^

Our study found a significant correlation between hyponatremia and lung disease. The lung diseases for which our patients were tested were COPD, asthma, idiopathic pulmonary fibrosis, and bronchiectasis. Few studies have described a relationship between COPD or asthma and hyponatremia. Further studies enrolling larger populations are required to verify the existence of an association between hyponatremia and the lung diseases analyzed herein.

SIADH secondary to amiodarone might be underdiagnosed in emergency and trauma centers. We believe that elderly patients on amiodarone or antidepressants should have serum sodium levels measured not only upon admission, but also during regular visits with their physicians. Additional attention is required in the presence of the following triads of findings: falls in the elderly/hyponatremia/amiodarone and falls in the elderly/hyponatremia/antidepressants. When these triads are present, physicians or medical teams with expert knowledge on the matter should further investigate patients for SIADH.

Our study had its share of limitations. The cross-sectional nature of the study and the relative lack of data - only 69 of 168 elderly patients with fragility fractures were included - decreased the sample space. Additionally, the study was carried out in only one regional private tertiary referral hospital.

More studies are needed to assess a possible correlation and the causal links between fragility fractures of the proximal femur and hyponatremia in elderly individuals. Our study found associations between some comorbidities and prescribed medication. Significant associations were found with amiodarone and antidepressants (SSRIs, SNRIs, mirtazapine, and trazodone). In the realm of comorbidities, lung diseases in general (asthma, COPD, idiopathic pulmonary fibrosis, and bronchiectasis) had a relevant role.
